# Special low protein foods for phenylketonuria: availability in Europe and an examination of their nutritional profile

**DOI:** 10.1186/s13023-015-0378-7

**Published:** 2015-12-22

**Authors:** Maria João Pena, Manuela Ferreira Almeida, Esther van Dam, Kirsten Ahring, Amaya Bélanger-Quintana, Katharina Dokoupil, Hulya Gokmen-Ozel, Anna Maria Lammardo, Anita MacDonald, Martine Robert, Júlio César Rocha

**Affiliations:** Centro de Genética Médica Doutor Jacinto de Magalhães, CHP EPE, Praça Pedro Nunes, 88, 4099-028 Porto, Portugal; Unit for Multidisciplinary Research in Biomedicine, Abel Salazar Institute of Biomedical Sciences, University of Porto-UMIB/ICBAS/UP, Porto, Portugal; Beatrix Children’s Hospital, University of Groningen, University Medical Center’ Groningen Netherlands, Groningen, Netherlands; Department of PKU, Kennedy Centre, Glostrup, Denmark; Unidad Enfermedades Metabolicas Servicio de Pediatria Hospital Ramon y Cajal, Madrid, Spain; Department of Metabolism and Nutrition, Dr. von Hauner Children’s Hospital, University of Munich, Munich, Germany; Department of Nutrition and Dietetics, Hacettepe University, Ankara, Turkey; Department of Pediatrics, San Paolo Hospital University of Milan, Milan, Italy; The Children’s Hospital, Birmingham, UK; Nutrition and Metabolism Unit, Hôpital Universitaire des Enfants Reine Fabiola, Brussels, Belgium; Faculdade de Ciências da Saúde, Universidade Fernando Pessoa, Porto, Portugal; Center for Health Technology and Services Research (CINTESIS), Porto, Portugal

**Keywords:** Phenylketonuria, Special low protein foods, Availability, Nutritional composition, Europe

## Abstract

**Background:**

Special low protein foods (SLPF) are essential in the nutritional management of patients with phenylketonuria (PKU). The study objectives were to: 1) identify the number of SLPF available for use in eight European countries and Turkey and 2) analyse the nutritional composition of SLPF available in one of these countries.

**Methods:**

European Nutritionist Expert Panel on PKU (ENEP) members (Portugal, Spain, Belgium, Italy, Germany, Netherlands, UK, Denmark and Turkey) provided data on SPLF available in each country. The nutritional composition of Portuguese SLPF was compared with regular food products.

**Results:**

The number of different SLPF available in each country varied widely with a median of 107 [ranging from 73 (Portugal) and 256 (Italy)]. Food analysis of SLPF available from a single country (Portugal) indicated that the mean phenylalanine content was higher in low protein baby cereals (mean 48 mg/100 g) and chocolate/energy bars/jelly (mean 41 mg/100 g). The energy content of different foods from a sub-group of SLPF (cookies) varied widely between 23 and 96 kcal/cookie. Low protein bread had a high fat content [mean 5.8 g/100 g (range 3.7 to 10)] compared with 1.6 g/100 g in regular bread. Seven of the 12 SLPF sub-groups (58 %) did not declare any vitamin content, and only 4 (33 %) identified a limited number of minerals.

**Conclusions:**

Whilst equal and free access to all SLPF is desirable, the widely variable nutritional composition requires careful nutritional knowledge of all products when prescribed for individual patients with PKU. There is a need for more specific nutritional standards for special low protein foods.

## Background

Early diagnosis and treatment of patients with phenylketonuria (PKU) prevents major neuro-cognitive deficits [1]. This is mainly achieved through the prescription of a phenylalanine (Phe) restrictive diet, consisting of natural protein and Phe restriction, together with L-amino acids supplements and special low protein foods (SLPF) [2]; the latter also used in other inborn errors of amino acid metabolism. The function of SLPF in the PKU diet is to provide energy and variety in the diet [2]. In PKU much emphasis has been placed on the role and dose of L-amino acids but little attention is given to the nutritional or energy profile of SLPF [3]. It is estimated that the patient’s SLPF intake will depend on disorder severity, providing around 50 % of the total energy intake in the most severe forms of the disorder. Milder phenotypes, with a higher natural protein tolerance are likely to be less dependent on their use [3].

SLPF have a low protein and Phe content but their micronutrient composition is unclear. In a normal diet, healthy energy food sources commonly provide other macro and micronutrients. In direct contrast, SLPF micronutrient fortification is uncommon and it is assumed that the majority of vitamins and minerals are provided from L-amino acids supplemented with micronutrients [4, 5].

SLPF are regulated by the European legislation ‘Foods for Special Medical Purposes’ (Commission Directive 1999/21/EC of 25 March 1999; amended in Directive 2006/141/EC). This Directive sets out rules for the composition and labeling of foods that are specifically formulated, processed and intended for the dietary management of diseases, disorders or medical conditions of individuals who are being treated under medical supervision. The nutritional substances that may be used in the manufacture of foods for special medical purposes are also outlined in legislation: Commission Regulation (EC) No 953/2009 [6]. In addition, all Foods for Special Medical Purposes have to follow the European Food Information to Consumers Regulation No 1169/2011 and Regulation No 609/2013 which is only just being enforced in many countries. Manufacturers must provide information concerning the energy value and principal nutrients contained in such foods but only have to declare the vitamins and/or minerals if they are present in “significant amounts”.

The objectives of this study were to identify the number of SLPF accessible for use in eight European countries and Turkey and to study the differences in the nutritional composition of SLPF available in one country (Portugal).

## Methods

In July 2014, comprehensive nutritional composition data of SLPF suitable for PKU and available in Portugal was collected from dietary companies or from SLPF packaging labels. Excel product nutritional composition databases with the numbers and types of SLPF were created.

Products were organized in sub-groups: baby cereals, bread, breakfast cereals, cakes/mix cakes/pancake mix, chocolate/energy bars/jelly, cookies, flour, ice cream, milk replacers, pasta, rice and savoury foods. Nutritional composition was expressed in weight of product per 100 g or 100 mL. Vitamin and mineral profiles were compared with regular foods, matched for food type. Their nutritional composition was obtained from the Portuguese Food Composition Table (available at www.insa.pt) (exception was the micronutrient composition of regular baby cereals which was collected from respective food companies).

Participants from the European Nutritionist Expert Panel on PKU (ENEP) [a group of dietitians/medical doctors from PKU centers in Europe (Portugal, Spain, Belgium, Italy, Germany, Netherlands, UK, Denmark) and Turkey] were invited to provide a list of SLPF available in their countries.

## Results

### Number of SLPF available in Europe and Turkey

In Italy there were over twice as many SLPF (*n* = 256) available compared with other countries, although in 4 countries (Spain, Netherlands, Turkey and Denmark) (Table 1) the exact number of products available was unknown. Portugal had the least number of SLPF (*n* = 73) (Table 1).

**Table 1:** Number of different SLPF available in each country

Product	PT	SP	BE	IT	GER	NL	TK	UK	DK
SLPF	73	NI	92	256	94	NI	NI	121	NI

PT = Portugal; SP = Spain; BE = Belgium; IT = Italy; GER = Germany; NL = Netherlands; TK = Turkey; UK = United Kingdom; DK = Denmark; SLPF = Special low protein foods; NI = no information

### Nutritional composition of Portuguese SLPF

All Portuguese SLPF contained a mean Phe content of less than 50 mg/100 g [mean 21 mg (6 mg in milk replacers; 48 mg in baby cereals)], which is an acceptable level for a SLPF for PKU (Table 2). Energy content varied widely between different SLPF sub-groups (Table 2). The majority of SLPF sub-groups (with the exception of milk replacers) contained energy content ranging from 305 to 478 Kcal/100 g of product. Milk replacers had a mean energy content of 99 kcal/200 mL, whereas cookies contained a mean energy content of 51 kcal per 11 g unit. For 14 different cookies available in Portugal, the energy content varied between 23 and 96 kcal per unit. The SLPF energy content was predominantly from carbohydrate (CHO) and fat sources. SLPF with higher fat content had the highest energy content (Table 2).

**Table 2:** Nutritional composition of SLPF available in Portugal, according to sub-groups

SLPF	Usual portion	Phe (mg)	Protein (g)	Fat (g)	CHO (g)	Energy (Kcal)
Baby Cereals	30 g	48 [4–70]	1.3 [0.2–1.8]	10.0 [0.5–14.8]	83.0 [77.1–95.0]	415 [381–449]
Bread	50 g	29 [10–65]	0.8 [0.5–1.3]	5.8 [3.7–10.0]	61.3 [50.0–82.3]	305 [236–412]
Breakfast Cereals	30 g	13 [5–31]	0.5 [0.2–1.0]	1.1 [0.7–1.9]	92.2 [90.5–93.6]	381 [370–387]
Cakes/Mix Cakes/Pancake Mix	50 g	11 [4–30]	0.4 [0.2–0.9]	5.2 [0.2–15.2]	77.6 [58.0–88.2]	362 [341–372]
Chocolate/Energy Bars/Jelly	unit	41 [10–90]	1.2 [0.2–2.5]	18.5 [1.0–33.6]	67.6 [42.6–93.3]	441 [377–548]
Cookies	unit	14 [2–34]	0.4 [0.1–0.8]	19.1 [1.5–49.4]	75.1 [48.3–87.7]	478 [395–639]
Flour	*variable*	13 [10–15]	0.3 [0.3–0.4]	0.8 [0.4–1.1]	86.6 [82.8–89.6]	357 [344–372]
Ice Cream	unit	27 [14–43]	0.7 [0.3–1.1]	4.5 [3.3–5.2]	85.6 [82.0–88.9]	386 [376–394]
Milk replacers	200 mL	6 [0.0–10]	0.2 [0.0–0.4]	2.7 [2.0–3.8]	6.1 [4.8–8.1]	49 [40–66]
Pasta	40 g	12 [11–13]	0.4 [0.2–0.5]	0.8 [0.6–1.2]	86.2 [85.4–87.4]	355 [348–363]
Rice	40 g	13	0.4	0.8	88.9	365
Savoury Foods	unit	26 [0–90]	0.7 [0.0–2.1]	16.2 [0.0–39.5]	40.5 [0.0–88.4]	323 [0–577]

CHO = Carbohydrate; Phe = Phenylalanine; SLPF = Special low protein foods

Nutritional composition data is presented in mean [range] per 100 g / 100 mL of each SLPF sub-group

Within the SLPF sub-group of bread, the mean fat content was 5.8 g/100 g with two products containing almost 10 g/100 g, which is in contrast with the content usually found in regular bread [1.6 g/100 g (Table 3)]. The energy contribution from CHO was also considerably higher. The CHO content of SLPF cookies was higher compared with regular cookies (Table 3).

**Table 3:** Macronutrient and energy content of SLPF and regular foods available in Portugal

SLPF/regular foods	Protein (g)		Fat (g)		CHO (g)		Energy (Kcal)	
	SLPF	Regular foods	SLPF	Regular foods	SLPF	Regular foods	SLPF	Regular foods
Baby Cereals	1.3 [0.2–1.8]	9.7 [5.9–16.5]	10.0 [0.5–14.8]	3.4 [1.1–7.8]	83.0 [77.1–95.0]	59.0 [66.8–89.4]	415 [381–449]	397 [393–403]
Bread	0.8 [0.5–1.3]	7.7 [5.3–11.2]	5.8 [3.7–10.0]	1.6 [0.8–2.3]	61.3 [50.0–82.3]	55.6 [37.2–71.6]	305 [236–412]	274 [185–360]
Breakfast Cereals	0.5 [0.2–1.0]	13.8 [13.5–14.0]	1.1 [0.7–1.9]	4.2 [2.5–5.8]	92.2 [90.5–93.6]	65.4 [61.7–69.0]	381 [370–387]	360 [360–360]
Cakes/Mix Cakes/Pancake Mix	0.4 [0.2–0.9]	5.8 [4.2–7.4]	5.2 [0.2–15.2]	28.7 [26.4–31.0]	77.6 [58.0–88.2]	42.4 [37.8–47.0]	362 [341–372]	451 [445–456]
Chocolate/Energy Bars/Jelly	1.2 [0.2–2.5]	47.5 [8.0–87.0]	18.5 [1.0–33.6]	17.0 [0.1–33.9]	67.6 [42.6–93.3]	26.6 [0.0–53.1]	441 [377–548]	448 [349–546]
Cookies	0.4 [0.1–0.8]	9.1 [7.3–10.8]	19.1 [1.5–49.4]	15.2 [12.2–17.8]	75.1 [48.3–87.7]	65.8 [61.0–72.0]	478 [395–639]	442 [436–451]
Flour	0.3 [0.3–0.4]	8.4 [7.8–9.0]	0.8 [0.4–1.1]	1.2 [1.1–1.3]	86.6 [82.8–89.6]	75.2 [74.3–76.0]	357 [344–372]	354 [347–361]
Ice Cream	0.7 [0.3–1.1]	2.0 [0.4–3.6]	4.5 [3.3–5.2]	5.5 [0.0–10.9]	85.6 [82.0–88.9]	27.2 [21.7–32.6]	386 [376–394]	164 [130–198]
Milk replacers	0.2 [0.0–0.4]	3.4 [3.3–3.4]	2.7 [2.0–3.8]	0.9 [0.2–1.6]	6.1 [4.8–8.1]	9.8 [4.9–4.9]	49 [40–66]	41 [34–47]
Pasta	0.4 [0.2–0.5]	13.2 [12.4–13.9]	0.8 [0.6–1.2]	2.5 [1.8–3.1]	86.2 [85.4–87.4]	68.8 [67.6–70.0]	355 [348–363]	358 [354–361]
Rice	0.4	6.7	0.8	0.4	88.9	78.1	365	352
Savoury Foods	0.7 [0.0–2.1]	25.1 [20.2–30.0]	16.2 [0.0–39.5]	10.4 [6.8–14.0]	40.5 [0.0–88.4]	0.1 [0.0–0.2]	323 [0–577]	195 [142–247]

CHO = Carbohydrate; SLPF = Special low protein foods

Nutritional composition data is presented in mean [range] per 100 g / 100 mL of each SLPF sub-group and corresponding regular Portuguese foods

Information regarding age suitability of SLPF was not available on packaging for all products, although the majority contained this information. In general, SLPF food labels provided unclear information about fat and CHO quality/sources. The micronutrient composition of SLPF was lower compared with regular-matched foods in terms of vitamin and mineral profiles (Tables 4 and 5). Considering all the 73 Portuguese SLPF analysed, 50 (69 %) of them stated only the mineral content on the label, 16 (22 %) of them gave no information about the vitamin and mineral profile and just 7 (10 %) products described the presence of vitamin and minerals on the label. In 7 of 12 SLPF sub-groups (58 %) the presence of any vitamin was not labelled on the packaging. For minerals, labelling was more common and in 8 of the 12 SLPF sub-groups (67 %), 100 % of the food items identified minerals on the food labelling.

**Table 4:** Vitamin profile of SLPF sub-groups in comparison with regular-matched foods available in Portugal

SLPF			Regular-matched foods		
Food item (N)	% of foods with vitamin content identified on food label	Vitamins	Food item (N)	% of foods with vitamin content identified on food label	Vitamins
Baby Cereals (*n* = 4)	50 %	A, D, E, K, C, B1, B2, B3, B5, B6, B9, B12. *	Baby Cereals (*n* = 3)	100 %	A, D, E, K, C, B1, B2, B3, B5, B6, B9, B12.
Bread (*n* = 9)	0 %	*	Bread (*n* = 4)	100 %	E, B1, B2, B3, B6, B9.
Breakfast Cereals (*n* = 4)	50 %	A, D, E, K, C, B1, B2, B3, B5, B6, B9, B12. *	Breakfast Cereals (*n* = 2)	100 %	E, B1, B2, B3, B6, B9.
Cakes/Mix Cakes/Pancake Mix (*n* = 6)	0 %	*	Cakes/Mix Cakes/Pancake Mix (*n* = 2)	100 %	A, D, E, B1, B2, B3, B6, B9, B12.
Chocolate/Energy Bars/Jelly (*n* = 5)	20 %	E, B1. *	Chocolate/Energy Bars/Jelly (*n* = 2)	50 %	A, E, B1, B2, B3, B6, B9, B12.
Cookies (*n* = 14)	7 %	B1, B2, B3, B6. *	Cookies (*n* = 4)	100 %	E, B1, B2, B3, B6, B9.
Flour (*n* = 3)	33 %	B1, B2, B3, B6. *	Flour (*n* = 2)	100 %	E, B1, B2, B3, B6, B9.
Ice Cream (*n* = 3)	0 %	*	Ice Cream (*n* = 2)	50 %	A, D, E, C, B1, B2, B3, B6, B9, B12.
Milk replacers (*n* = 4)	0 %	*	Milk (*n* = 2)	100 %	A, D, E, B1, B2, B3, B6, B9, B12.
Pasta (*n* = 13)	0 %	*	Pasta (*n* = 2)	100 %	D, E, B1, B2, B3, B6, B9.
Rice (*n* = 1)	0 %	*	Rice (*n* = 1)	100 %	E, B1, B2, B3, B6, B9.
Savoury Foods (*n* = 7)	0 %	*	Savoury Foods (*n* = 2)	100 %	D, E, B1, B2, B3, B6, B9, B12.

*All other products/All products either have nil content or no information in label

**Table 5:** Mineral profile of SLPF sub-groups in comparison with regular-matched foods available in Portugal

SLPF			Regular-matched foods		
Food item (N)	% of foods with mineral content identified on food label	Minerals	Food item (N)	% of foods with mineral content identified on food label	Minerals
Baby Cereals (*n* = 4)	100 %	Ca, Fe, Na, K, Zn, Se, Cu.	Baby Cereals (*n* = 3)	100 %	Ca, Fe, Na, K, Zn, I.
Bread (*n* = 9)	44 %	Ca, Na, K. *	Bread (*n* = 4)	100 %	Ca, Fe, Na, K, P, Mg, Zn.
Breakfast Cereals (*n* = 4)	100 %	Fe, Na, K.	Breakfast Cereals (*n* = 2)	100 %	Ca, Fe, Na, K, P, Mg, Zn.
Cakes / Mix Cakes / Pancake Mix (*n* = 6)	100 %	Ca, Na, K.	Cakes / Mix Cakes / Pancake Mix (*n* = 2)	100 %	Ca, Fe, Na, K, P, Mg, Zn.
Chocolate / Energy Bars / Jelly (*n* = 5)	100 %	Ca, Na, K.	Chocolate / Energy Bars / Jelly (*n* = 2)	100 %	Ca, Fe, Na, K, P, Mg, Zn.
Cookies (*n* = 14)	86 %	Ca, Fe, Na, K. *	Cookies (*n* = 4)	100 %	Ca, Fe, Na, K, P, Mg, Zn.
Flour (*n* = 3)	100 %	Na, K.	Flour (*n* = 2)	100 %	Ca, Fe, Na, K, P, Mg, Zn.
Ice Cream (*n* = 3)	100 %	Ca, Na, K.	Ice Cream (*n* = 2)	100 %	Ca, Fe, Na, K, P, Mg, Zn.
Milk replacers (*n* = 4)	100 %	Ca (in 50 %), Na, K.	Milk (*n* = 2)	100 %	Ca, Fe, Na, K, P, Mg, Zn.
Pasta (*n* = 13)	23 %	Na, K. *	Pasta (*n* = 2)	100 %	Ca, Fe, Na, K, P, Mg, Zn.
Rice (*n* = 1)	100 %	Na, K.	Rice (*n* = 1)	100 %	Ca, Fe, Na, K, P, Mg, Zn.
Savoury Foods (*n* = 7)	71 %	Ca, Fe, Na, K. *	Savoury Foods (*n* = 2)	100 %	Ca, Fe, Na, K, P, Mg, Zn.

*All other products/All products either have nil content or no information in label

## Discussion

In PKU, a varying choice and availability of SLPF may disadvantage some European patients in their ability to achieve acceptable metabolic control. It was clear from this study, that there was no uniform availability of SLPF in eight European countries and Turkey, and in some countries there was limited knowledge and central ‘control’ about the range and type of products available. Different government policies and reimbursement strategies clearly contribute to this disparity [7–9].

A higher availability of SLPF is considered a pivotal part of a Phe-restricted diet. While their ingestion satisfies energy needs, they also help support “free” amino acids anabolism, improve dietary adherence and thereby help maintain blood Phe control within target ranges [4]. More investigation is needed in order to understand if availability of a higher and wider range of SLPF optimizes dietary adherence and contributes to improved nutritional status [10].

The energy content of all SLPF is important and clearly justifies special attention when prescribing to patients in different clinical situations. In our opinion, the results presented here clearly justify improved labelling that would lead to better food choices. Considering the recent interest of nutritional status in the PKU management, other aspects of nutrition should be considered beyond blood Phe control [11]. Although similar to the general population, overweight is a concern in PKU, especially in older females [3] with poor Phe control. Dietary intake also clearly influences cardiometabolic markers and more detailed research is needed to understand if differences found between patients and controls have some origin in different dietary patterns [12]. When lipid and CHO compositions were compared with regular foods, we found higher contributions in 58 and 92 % of SLPF subgroups, respectively. These data, together with the fact that in 75 % of the SLPF sub-groups the energy content was higher than in regular foods, underlines the need for careful nutritional prescription and monitoring.

Another common feature of SLPF is lack of label micronutrient information when compared with regular matched-foods. Although micronutrients are mainly consumed through L-amino acid supplements [13, 14], it is important that the nutritional profile is fully identified on the label. Also SLPF should contain a warning indicating that their nutritional profile does not replicate regular foods because patients, caregivers and health professionals may assume they provide other nutrients other than energy. At present, there are no detailed studies outlining their full nutritional contribution to a low Phe diet.

PKU is a chronic disorder and regular nutritional education is required [15]. It is essential that health professionals provide guidance on the amount of SLPF that should be prescribed in different clinical situations in PKU (e.g. children, pregnancy, overweight/obesity, with and without Sapropterin treatment). In order to optimize dietary prescription and to prevent nutritional status imbalances like overweight/obesity, the Portuguese PKU center adopted a color system in order to categorize SLPF, based on its nutritional profile. This analogy with the traffic light colors has been adopted on regular food labeling in order to keep the consumer alerts about the nutritional composition of food, mainly in respect with fat (especially saturated fat), salt and added sugars per 100 g (available at http://www.food.gov.uk).

## Conclusions

The difference found in the SLPF availability within Europe and Turkey is relevant and may contribute to difference in patient ability to maintain acceptable metabolic control across Europe. It is important that industry ensure a high quality of SLPF in respect to taste, acceptability and appearance to help improve patient adherence with diet therapy. However, it is also important that manufacturers increase the detail about nutritional composition and product labelling, although the European Food Information to Consumers Regulation No 1169/2011 should help improve the quality of information provided in the future. A careful analysis of nutritional profile of all products is desirable in order to better match the nutrient needs of each patient.

## Abbreviations

CHO: Carbohydrate
ENEP: European Nutritionist Expert Panel on PKU
Phe: Phenylalanine
PKU: Phenylketonuria
SLPF: Special Low Protein Foods
